# Quality of an Assistive Technology Web Application for Primary Care Physicians Serving Older Adults: Concurrent Mixed Methods Study

**DOI:** 10.2196/69645

**Published:** 2025-10-22

**Authors:** Elsa M Orellano-Colón, Wency Bonilla-Díaz, Radamés Revilla-Orellano, Jesús Mejías-Castro, Abiel Roche-Lima

**Affiliations:** 1 Medical Sciences Campus University of Puerto Rico San Juan Puerto Rico; 2 Huertas College Caguas Puerto Rico; 3 University of Puerto Rico at Humacao Humacao Puerto Rico

**Keywords:** aged, assistive technology, Latino, mobile applications, physically disabled, primary care physicians, usability testing

## Abstract

**Background:**

Older Latinos living in Puerto Rico experience significantly higher rates of functional disabilities (FDs; 1093/87,300, 27.8%) compared to older adults in the continental United States (755,685/57,913,200, 13.3%). While assistive technologies (ATs) can improve daily function and support aging in place, primary care physicians (PCPs), who are essential in addressing FDs resulting from chronic diseases, often lack knowledge about AT devices and services. The Mi Guía de Asistencia Tecnológica (MGAT; My Assistive Technology Guide) web application was empirically developed to address this gap by providing comprehensive information and videos about AT devices for older adults with functional difficulties in daily living activities.

**Objective:**

This study aimed to assess the quality of MGAT among PCPs and describe their experiences using the app to increase access to AT for older Latinos.

**Methods:**

A total of 10 PCPs participated in this usability project, receiving MGAT training before a 30-day implementation period. A concurrent mixed methods design was used, combining quantitative data from the User Version of the Mobile User Application Rating Scale (uMARS) and qualitative insights from semistructured individual interviews. The analysis included descriptive statistics and a directed content analysis.

**Results:**

The MGAT received high overall objective quality ratings on uMARS (mean 4.06, SD 1.05). Among subdomains, information scored highest (mean 4.60, SD 0.51), followed by functionality (mean 4.20, SD 0.63), aesthetics (mean 4.00, SD 0.82), and engagement, which scored lowest (mean 3.34, SD 1.51). Subjective quality ratings were also favorable, with a mean score of 3.93 (SD 1.19), with recommending the app to others scoring the highest (mean 4.70, SD 0.48) and willingness to pay for the app the lowest (mean 3.11, SD 1.90). Perceived impact received the highest score across all domains (mean 4.82, SD 0.39), with behavior change scoring the highest (mean 5.82, SD 0) and awareness scoring the lowest (mean 4.60, SD 0.52). Qualitative findings revealed that PCPs found MGAT entertaining and interesting, but wanted more customization and interactive features to boost engagement. They appreciated its ease of use and navigation, but noted the need for a stable internet connection. While the design was visually appealing, improvements to the color scheme and element sizes were suggested. Participants valued the high-quality information relevant to older adults but desired more specialized content for medical professionals. They were likely to recommend MGAT, though cost opinions varied. Most importantly, MGAT increased awareness of patient needs, expanded AT knowledge, and positively influenced intentions to recommend AT, ultimately facilitating patient access to AT.

**Conclusions:**

The high-quality and usefulness ratings suggest MGAT could be an effective tool for PCPs in managing older adults’ FDs. Future research should evaluate the effectiveness of MGAT in managing FDs among older adults.

## Introduction

Assistive technology (AT) devices, such as jar openers, sock aids, and canes, play a significant role in enhancing the daily functioning, safety performance in daily activities, and social participation of older adults, ultimately contributing to their well-being and ability to live independently in their homes and community [1-6]. Despite the benefits of AT, a previous qualitative research study indicated that Latino populations, particularly older adults in Puerto Rico, have limited access to these devices due to a lack of awareness and information about the available AT options [7,8]. This access gap can lead to functional deterioration and adverse health outcomes, including increased hospitalizations, reduced quality of life, and higher health care costs [9,10]. Furthermore, primary care physicians (PCPs) in Puerto Rico, defined as licensed health care professionals who provide medical services in areas such as general medicine, internal medicine, family medicine, and geriatrics, may also lack awareness of the AT resources available to their patients. This is critical since information on acquiring AT is a key factor influencing its usage among older adults [11].

PCPs serve as the first point of contact in the health care system and play a crucial role in addressing the functional limitations faced by older adults with chronic diseases. Since functional ability is essential for healthy aging [12], it is crucial for PCPs to have comprehensive knowledge of AT devices and services. This knowledge enables PCPs to effectively support older adults in maintaining their function, independence, and quality of life, which are essential to healthy aging. In addition, it allows PCPs to make informed decisions about their patients’ needs and facilitate access to appropriate resources [11].

The web application, Mi Guía de Asistencia Tecnológica (MGAT; My Assistive Technology Guide), was previously developed to address the information gap about AT for older Hispanics in Puerto Rico [13]. The content of the MGAT was determined based on the evidence of the need for AT devices in older Latinos living in Puerto Rico obtained from 2 quantitative descriptive studies [14,15]. The first quantitative study was initially conducted with a purposive sample of 60 independent-living Hispanic adults aged 70 years or older, residing in urban and rural areas of Puerto Rico. These individuals had physical functional disabilities (FDs) but no cognitive impairments [14]. Participants reported on approximately 50 AT devices for activities of daily living (ADLs) and instrumental activities of daily living, categorizing them into devices they used and those they did not use but were willing to consider using. A second cross-sectional study involved 211 independently living older Latinos aged 65 years or older, without cognitive impairments or FDs, who were randomly recruited from low-income communities in San Juan, Puerto Rico [15]. This study identified the most common physical disabilities using the Patient-Reported Outcomes Measurement Information System Physical Function Short Form. In addition, 2 qualitative studies, one with the purposive sample [14] and another with the randomly selected sample [7], explored barriers to AT device usage among 83 older Latino adults. Modifiable barriers identified in these studies informed both the selection of AT devices and the associated educational content provided for each device.

MGAT offers user-friendly access to detailed information about 94 AT devices in 8 categories of daily activities: mobility, self-care, bathing, dressing, food preparation, home management, medication management, and home safety. It incorporates visual aids and simple navigation to enhance its usability in providing information about AT devices to a variety of users.

MGAT has been studied to evaluate its quality and explore older Latinos with physical disabilities' experiences with it [16]. Using a mixed methods design, the study involved 12 participants who were trained to use the MGAT, then used it for 30 days, and finally provided feedback through quantitative ratings obtained from the Spanish version of the User Mobile Application Rating Scale (uMARS) [17] and semistructured interviews. The results showed that the MGAT was highly rated for both objective and subjective quality, with means of 3.99 and 4.13 (out of a maximum of 5), respectively. Qualitative findings indicated that the MGAT was accessible, usable, desirable, credible, useful, and valuable for increasing older Latinos’ knowledge and autonomy regarding AT. Overall, the MGAT showed potential to improve AT awareness and adoption among older adults, addressing a significant barrier to AT use. This shows promise as a valuable resource for PCPs in managing FDs among older adults, but further research is needed to understand their perspectives on its usability and integration into clinical workflows. This gap highlights the importance of assessing PCPs’ experiences with the MGAT, as they are the primary point of contact for older adults and play a critical role in promoting access to AT.

A mixed method research design that combines quantitative assessments using tools such as the uMARS [17] with qualitative insights from user interviews provides a comprehensive understanding of user experiences and perceptions [18]. This approach captures both objective usability issues and subjective perceptions to specifically address PCPs’ unique needs around clinical decision support in AT. Mixed methods will provide comprehensive actionable insights that neither quantitative nor qualitative approaches alone could deliver, resulting in an app that meets both technical usability standards and seamlessly integrates into PCPs’ demanding clinical workflows. Furthermore, this integrated approach not only identifies strengths but also highlights areas for improvement, ensuring that health applications like MGAT effectively support both health care providers and their patients in accessing AT resources.

This study aimed to assess the quality of the MGAT among PCPs and describe their experiences using it in a real-world setting in supporting consultations with older adults. By providing culturally relevant information on AT options, MGAT can help PCPs assess patients’ needs more effectively and educate them about available solutions. However, evaluating the quality and usability of such health applications before widespread implementation is crucial to guide health care professionals in making informed recommendations [19,20].

## Methods

### Study Design

**Figure 1:**
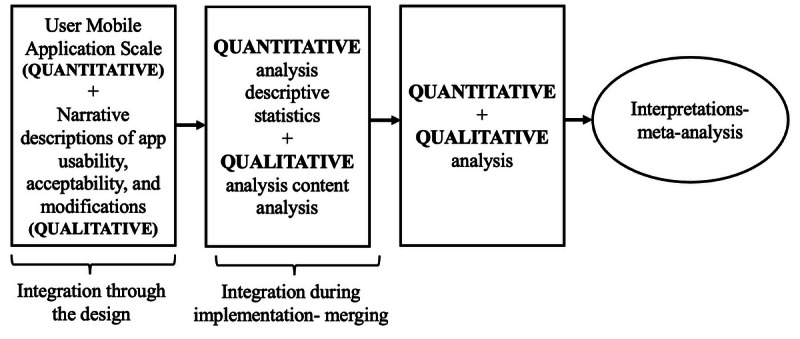
Integrated concurrent mixed methods design for MGAT (Mi Guía de Asistencia Tecnológica, My Assistive Technology Guide) evaluation.

To assess the quality and user experiences with the MGAT, 10 PCPs were encouraged to use the web application for 30 days. At the end of this usage period, the researchers used a concurrent mixed method design, which consisted of simultaneously collecting quantitative and qualitative data within a single study phase (see Figure 1). We selected this design to achieve the study’s aims, allowing the qualitative phase to enrich the quantitative results and thereby providing a comprehensive understanding of the quality and user experiences with the MGAT web application from the PCPs’ perspectives. In this study, quantitative data were obtained measuring the quality of the MGAT web application using the uMARS tool [17]. Qualitative data were collected through semi-structured interviews conducted concurrently after participants responded to each item of the uMARS.

Integration of mixed methods occurred at 3 points, as described by Fetters et al (see Figure 1) [21]. First, integration through the design occurred during the data collection phase by collecting quantitative and qualitative data simultaneously in the same interview at the end of the MGAT web application trial period. The qualitative in-depth interview questions were informed by data provided in the quantitative section. Second, we used the merging approach for the integration during implementation. During the merging approach, we brought together quantitative and qualitative data to expand on the uMARS items rated with a 3 or less (disagree or strongly disagree). Finally, quantitative and qualitative data were integrated during interpretation through meta-inference using a narrative integration approach. This method allowed us to combine numerical data from the uMARS with thematic insights from interviews, providing a comprehensive understanding of the study’s findings. By merging these data types, we gained a richer perspective on the usability and quality of the MGAT web application.

Karagianni’s optimized honeycomb model [22], originally developed by Morville [23], was the theoretical perspective that guided data collection, analysis, interpretation, and discussion of the study results. The model was chosen due to its applicability to analyzing the user experience perspective during the application’s design. It consists of 7 facets used to analyze the web application user experience perspective: findable, accessible, usable, desirable, credible, useful, and valuable. These facets are grouped into 3 parts, reasoning about how the user “Feel, Think and Use” the product by connecting the 7 facets.

### Intervention

**Figure 2:**
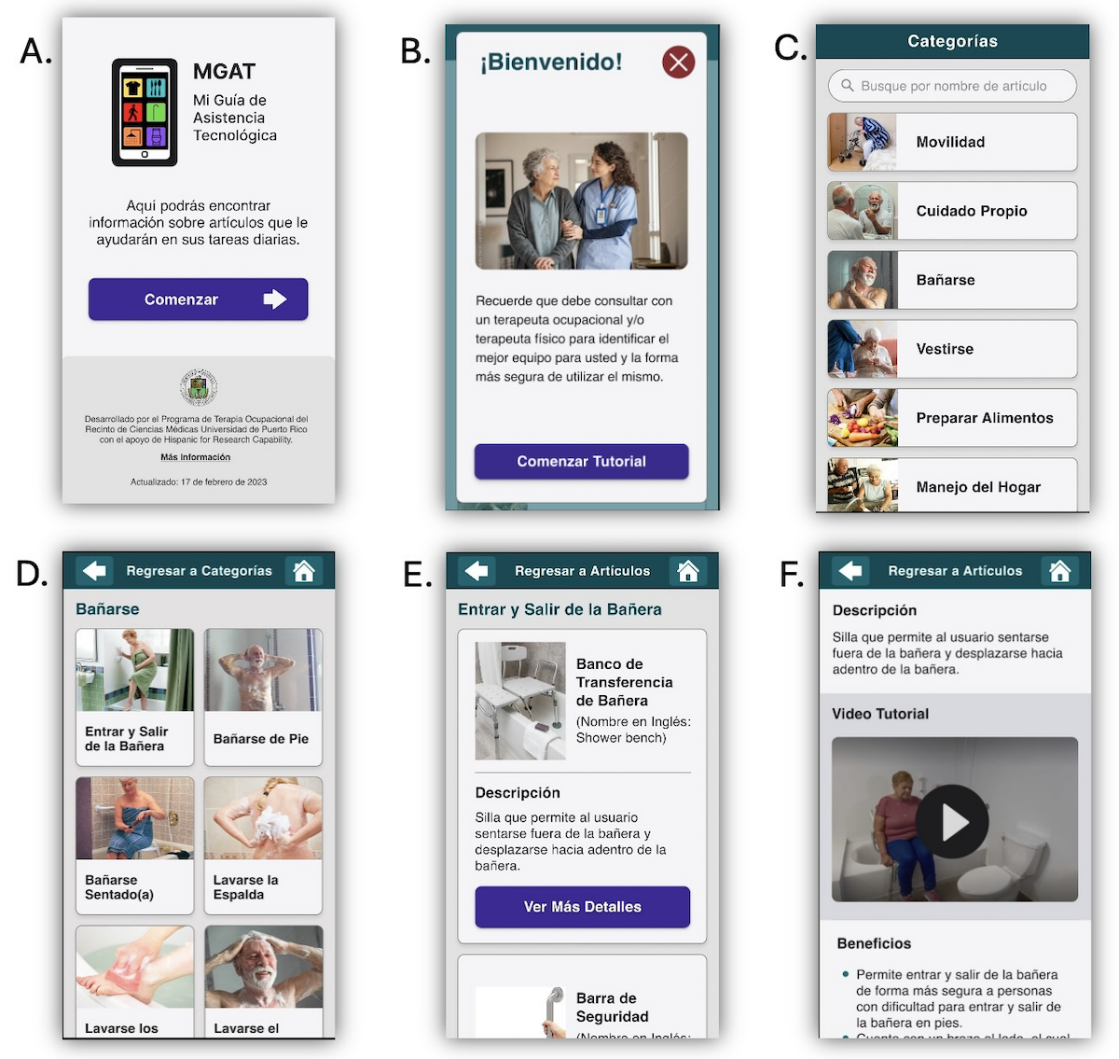
Screenshots of the MGAT (Mi Guía de Asistencia Tecnológica, My Assistive Technology Guide) web application.

The MGAT web application had been developed in a previous study to increase community-living older Latinos’ access to information about AT devices and services that could increase this population’s functional performance in everyday activities [13]. The content included in the MGAT had been determined based on the following: (1) evidence of AT device needs of older Latinos living in Puerto Rico found in previous studies [14,15]; (2) the Matching Person and Technology Model [24], which considered the person, technology, and environmental factors found in previous studies that influenced the use of AT among older Latinos from poor communities (eg, AT with aesthetic appearance [umbrella cane] for overcoming the stigma associated with the use of mobility devices); and (3) evidence-based guidelines and recommendations for designing health applications for older adults reported recently in the literature [25-29]. The MGAT had been designed with two fundamental characteristics (see Figure 2). First, the app was designed to be simple to use, regardless of the user’s familiarity with mobile apps, making it accessible to everyone. Second, it allowed the user to effectively navigate the MGAT through 8 different areas of ADL to finally learn about the available AT devices used to compensate for the FDs that older adults might have in each daily living activity. The user could explore AT devices within 8 broad areas of ADL in which older adults might exhibit functional difficulties using buttons with photos and text to represent each area (mobility, self-care, bathing, dressing, meal preparation, home management, and medication management). Each of these areas contained subcategories of activities of each activity (see Table 1).

**Table 1:** Categories and subcategories of the daily activities included in the MGAT web application.

Categories and subcategories		Assistive technology devices included in the web application (N=94), n (%)
**Mobility**		
	Getting in and out of bed	2 (2)
	Sitting and standing from chairs	2 (2)
	Walking	11 (13)
	Getting in and out of a car	5 (5)
	Sitting and standing from the toilet	6 (6)
**Self-care**		
	Brushing teeth	3 (3)
	Cutting fingernails	2 (2)
	Cutting toe nails	2 (2)
**Bathing**		
	Getting in and out of the shower	3 (3)
	Bathing standing up	3 (3)
	Bathing sitting down	3 (3)
	Washing back	1 (1)
	Washing feet	2 (2)
	Washing hair	1 (1)
**Dressing**		
	Putting on and taking off a shirt	1 (1)
	Buttoning shirt	1 (1)
	Putting on and taking off pants	2 (2)
	Putting on and taking off socks	4 (4)
	Putting on shoes	3 (3)
**Meal preparation**		
	Using utensils	2 (2)
	Pouring liquids	2 (2)
	Cutting food	2 (2)
	Opening jars	6 (6)
	Opening cans	4 (4)
	Opening bottles	2 (2)
**Home management**		
	Reaching objects	2 (2)
	Carrying objects	3 (3)
	Cleaning bathroom	1 (1)
	Cleaning floor	2 (2)
	Opening doors	1 (1)
**Medication management**		
	Opening medication containers	2 (2)
	Cutting pills	1 (1)
	Crushing pills	1 (1)
	Organizing pills	2 (2)
**Home safety**		
	Walking over carpets	2 (2)
	Walking in dark areas	1 (1)
	Medical alert systems	1 (1)

The MGAT web application features several key screens: an introductory screen that leads to the home page; a tutorial screen designed to guide users; a screen presenting categories of daily activities; a screen that lists specific activities within each selected category; a screen showcasing various assistive technology devices; and a final screen offering detailed information and instructional videos about these assistive technology devices.

In addition, the MGAT features instructional videos demonstrating older adults using AT devices. As an informational web application, the MGAT lacks interactive functionalities. It is designed to be compatible with any mobile device to enhance free access for users. While the MGAT offers valuable insights into AT devices, it does not guide the selection of the most appropriate device based on individual requirements. Consequently, all videos include a disclaimer: “The equipment shown in this video is only demonstrative. This video is solely for educational purposes. We recommend that you consult with your occupational therapist to evaluate your need for assistive technology devices.”

### Participants, Sampling, and Recruitment

Three researchers (an occupational therapist, a doctoral student in medicine, and a biology bachelor’s degree student) received 24-hour training from the principal investigator to recruit, collect, and analyze data. The principal investigator is an occupational therapist with extensive experience conducting mixed method study designs. These researchers recruited a purposive sample of 10 PCPs who deliver services to older adults with physical FDs, following the sampling guidelines proposed by Hwang and Salvendy [30]. Their “10 ± 2 rule,” based on a meta-analysis of usability studies, establishes this range as optimal for user testing, balancing statistical power with practical feasibility

This selection is further supported by Nielsen and Landauer’s [31] problem discovery model, which demonstrates that a sample of 10 participants typically identifies 95% of usability issues. In health care-specific usability testing, Kushniruk and Patel [32] found that a participant range of 8-12 is sufficient for detecting major usability concerns in clinical information systems. Our study methodology incorporated task-based scenarios aligning with Kushniruk and Patel’s [32] validated approaches for health care interface evaluation.

Furthermore, our findings demonstrated saturation in issue discovery, with no new critical usability concerns emerging in the final 2 participants, empirically confirming the adequacy of our sample size within this specific context. While larger samples may be required for broader qualitative research objectives, the chosen sample size effectively balances resource efficiency with comprehensive problem identification, ensuring a robust evaluation of the user interface.

Study sampling was carried out using purposive sampling, which involved an interactive process of selecting research subjects (based on their ability to elucidate a specific theme, concept, or phenomenon, in this case, the usability and acceptability of the AT web application). Individuals were recruited until the researchers perceived that no new themes or subthemes were emerging (ie, data saturation), a standard approach in qualitative research [33].

The inclusion criteria were (1) adults 21 years and older, and (2) being a PCP who provided health care to older Latinos with physical FD. PCPs could include general practitioners, family practice physicians, internal medicine physicians, or geriatricians. The exclusion criteria were PCPs who were 20 years or younger and those who did not provide health care services to older Latinos with physical function disabilities.

A total of 2 recruitment methods were used: (1) direct contact through PCPs’ telephone numbers in Puerto Rico, obtained from publicly available internet-based contact information, and (2) snowball sampling procedures. During direct contact, researchers with experience in occupational therapy and general medicine called potential participants and provided the PCPs with a complete explanation of the purpose of the study, the study procedures, and that their participation in the study was completely voluntary. If the PCP expressed interest in participating in this study, the researchers assessed if the PCP met the 2 inclusion criteria and proceeded, with eligible participants, to schedule their convenient day, time, and place to conduct the consent form procedures. On the day of the consent form procedures, the researchers first met individually with each PCP in a private place at their preferred location (such as the clinic) to provide them with a complete explanation of the consent form, including the purpose, procedures, risks, and benefits of the study. The researchers gave PCPs the opportunity to ask any questions related to this study. After addressing all PCP concerns related to this study, the researchers asked them to sign the consent form and begin their participation in this study.

### Data Collection Procedures

The researchers scheduled a baseline meeting with each PCP to fill out a sociodemographic questionnaire and provide them with training in using the MGAT. The download of the app took approximately 2 minutes, and the MGAT training took approximately 15 minutes. PCPs were trained using verbal instructions, demonstrations, and hands-on practice until they demonstrated competence in using the web application. Training ensured engagement and skill retention through real-time navigation, guided practice, and competency-based assessment. PCPs were required to demonstrate their ability to navigate and use the web application effectively. This included completing specific tasks and scenarios using the MGAT, such as identifying AT devices to compensate for difficulties in different daily activities. Researchers conducted informal assessments during the training sessions to evaluate PCPs' understanding of the MGAT’s functionalities and their ability to apply it in clinical settings. All PCPs had to successfully complete key tasks before integrating the web application into their practice. This structured approach ensured they were fully prepared to use the app effectively in patient care.

Afterward, PCPs were encouraged to use the MGAT app for 30 days while providing health care services to older adults with physical function difficulties performing ADL. At the end of the web application usage period, the researchers scheduled an individual face-to-face or videoconferencing meeting with each clinician in their preferred location and convenient time to collect quantitative data using the uMARS and qualitative data through in-depth semistructured interviews on the experience, usage, and acceptability of the MGAT. During the interview, the researchers also conducted follow-up questions concerning the uMARS items the PCPs rated with a score of three or less (some disagreement). The principal investigator (PI), an expert in mixed methods design, provided 8 hours of training to all members of the research team in conducting PCP recruitment and administration of the study measures. The data collection process was completed in one face-to-face or videoconferencing meeting. With the consent of PCPs the interviews were audio recorded with the consent of the PCPs, and field notes were taken to capture further details. The researchers produced verbatim transcripts of the audio-recorded interviews for subsequent analysis.

### Assessment Tools

All assessment tools were conducted in Spanish and administered by the researchers of this study using an interview format on the convenient day of the PCP and the preferred location under the supervision of the PI after data collection training.

#### Sociodemographic Data Questionnaire

The team developed this questionnaire to collect the self-reported sociodemographic data of the following PCPs: (1) age, (2) sex, (3) medical specialty, (4) years of experience working with older adults with FDs, (5) average of older adults with FD attended monthly, (6) work setting, (7) frequency of app usage for the provision of health care services, (8) medical practice identifying and addressing FDs of older adults’ FDs, and (9) types of assistive devices recommended to older adults with FD. This measure took approximately 10 minutes to complete and was administered before the MGAT usage trial period.

#### uMARS Spanish Version

The uMARS is a 29-item scale that provides comprehensive ratings of user experience and impressions of the mobile health application by assessing the quality of the application (objective and subjective) and perceived impact [34]. Each item has a custom wording that is appropriate for the aspect being assessed. Items use a common 5-point rating scale from 1 (inadequate) to 5 (excellent), such that higher scores represent a stronger impact of the app on that aspect of user cognition or potential behavior. The subjective quality and perceived impact of the MGAT were assessed on an individual basis per item. The English version of uMARS was cross-culturally adapted for the Spanish language using a 3-process design: cross-cultural adaptation, translation, and evaluation of statistical reliability and validity [17].

#### Interview Guide

The researchers in this study developed this guide to facilitate the discussion and collection of qualitative data from PCPs necessary to further assess the usability, acceptability, and modifications of the MGAT web application. The guide used open questions to obtain participants’ feedback on their experience using the MGAT and their recommendations to improve its usefulness for PCPs. Interview questions included the following: “How adept do you think you are at using the app? Did you experience any difficulty when using the app? How did you cope with these issues? What are the strengths and weaknesses of the app? How can you improve the app? How useful is this app in your health service delivery system to older adults with FDs? Will you use this app as part of your health care delivery services? Why?” Furthermore, follow-up questions were asked to explain those items of the uMARS with a score of 3 or less. The interviews took an average of 20 minutes and were administered at the end of the MGAT usage trial period.

### Data Analysis

#### Quantitative Data

Quantitative data from the sociodemographic questionnaire and uMARS were analyzed using descriptive statistics of the central tendency, presenting means (SDs) for continuous variables and frequencies with percentages for categorical variables. Microsoft Excel 2019 facilitated these statistical analyses.

#### Qualitative Data

A directed thematic content analysis was used to analyze the qualitative data obtained from semistructured interviews [35]. This directed qualitative content analysis was informed by the optimized honeycomb model to systematically analyze textual data through the predefined scales of uMARS. The analysis began with clear operational definitions for the objective quality, subjective quality, and perceived impact scales. The researchers deeply engaged with the data, identifying and highlighting text segments relevant to each scale and coding them accordingly. The research team held regular meetings to collaboratively interpret the findings. Data management was supported by QDA Miner Lite (version 3.0.2; Provalis Research), a qualitative data analysis software package.

To ensure rigor in the analysis, peer debriefing sessions were conducted with two researchers for each participant. In addition, an external audit was performed by a qualitative data analysis expert (the PI). This audit involved a comprehensive review of all interviews to confirm the interpretations and analyses carried out by the researchers.

#### Integration Through Analysis and Interpretation

After completing qualitative and quantitative analyses, the qualitative insights of individual interviews were merged with the quantitative findings of uMARS for a comprehensive evaluation and comparison. A narrative integration approach was used, interweaving qualitative and quantitative results on a theme-by-theme or concept-by-concept basis to present a more comprehensive understanding of the experiences of the participants. Both quantitative and qualitative analyses were conducted in Spanish, with specific quotes chosen to illustrate each theme translated into English by a bilingual researcher for inclusion in the paper. This method ensures that the original meaning and context of the quotes are maintained during translation while making the findings accessible to an English-speaking audience. This version maintains the original meaning while using different phrasing and structure.

### Ethical Considerations

The study was approved by the Institutional Review Board of the University of Puerto Rico, Medical Sciences Campus (protocol 2211060727). The study procedures complied with the Declaration of Helsinki. All PCP participants provided written informed consent before their enrollment in the study. The consent form detailed the study’s purpose, procedures, potential risks and benefits, the voluntary nature of participation, and the right to withdraw at any time without penalty. Participants were given ample opportunity to ask questions and have their concerns addressed before signing the consent form. PCPs received compensation for their time and effort invested in data collection. Each PCP was compensated US $50 for their time invested in data collection at the beginning of the study (during the first visit) and US $50 at the end of the 30-day trial period of the MGAT web application, for a total of US $100. The images of older adults displayed within the MGAT web application were sourced from publicly available images obtained from Google. Since MGAT is a prototype developed solely for nonprofit research purposes, these images are used under the fair use doctrine, which permits using copyrighted materials without obtaining permission from the copyright owner for noncommercial research activities.

## Results

### Sample Characteristics

A total of 13 eligible participants were approached. Three participants refused to participate due to time constraints. A total of 10 participants completed the semistructured interviews and surveys after the trial period of using the MGAT web application. The trial period ranged from 34 to 63 days among participants. Table 2 describes the characteristics of the participants, illustrating a diverse group of primary health care providers with a mean age of 45.7 (SD 14.2) years, ranging from 29 to 62 years. Most participants were women (n=6, 60%). Regarding medical specialties, most of the participants were geriatricians or fellows (n=6, 60%), followed by those in internal medicine (n=3, 30%). The participants had an average of 17.3 (SD 13.7) years of service to older adults, ranging from 2 to 33 years. On average, PCPs provided medical services to 42.5 (SD 40.2) older adults with FDs per month. The distribution in the workplace showed that most PCPs worked in private clinics (n=7, 70%), university hospitals (n=3, 30%), and assisted living facilities (n=3, 30%).

**Table 2:** Sociodemographic characteristics of study participants.

Participants characteristics						Total (N=10)
**Age (years)**						
				Mean (SD)	45.7 (14.2)	
				Range	29-67	
**Sex, n (%)**						
			Female		6 (60)	
			Male		4 (40)	
**Medical specialty **						
			Mean (SD)		3.93 (1.19)	
			Geriatrics or geriatric fellows, n (%)		6 (60)	
			Internal medicine, n (%)		3 (30)	
			Family medicine, n (%)		1(10)	
**Years of service to older adults**						
	Mean (SD)				17.3 (13.7)	
	Range				2-33	
**Older adult patients with FDs^a^seen by PCPs^b^ per month**						
		Mean (SD)				42.5 (40.2)
		Older adult patients with FDs seen by PCPs per month range (n)				20-150
Patients served using the MGAT^c^ range (monthly)						0-30
**Workplace, n (%)**						
			Private clinic		7 (70)	
			University hospital		5 (50)	
			Assisted living facility		3 (30)	
			University outpatient clinic		3 (30)	
			Primary health care clinic		1 (10)	
			Veteran hospital		1 (10)	

^a^FD: functional disability.

^b^PCP: primary care physician.

^c^MGAT: Mi Guía de Asistencia Tecnológica (My Assistive Technology Guide).

Table 3 provides an overview of the self-reported characteristics of the study participants. It includes information on their sex, age, medical specialty, years of service to older adults with physical function disabilities, and their workplaces. The participants represent a diverse group in terms of sex, age, and professional background, working in various health care settings such as private clinics, university hospitals, outpatient clinics, primary health care clinics, assisted living facilities, and veteran hospitals. The years of service among participants range widely, reflecting a broad spectrum of experience in the field.

**Table 3:** A snapshot of participants’ self-reported characteristics.

Participant ID (N=10)	Sex	Age (years)	Medical specialty	Years of service to older adults with physical function disabilities	Workplace
R1	Female	43	Internal medicine	15	University outpatient clinic
R2	Male	34	Internal medicine	3	Private clinic and primary health care clinic
R3	Female	54	Geriatrics	26	Private clinic, University hospital, and assisted living facility
R4	Male	67	Internal medicine	37	Private clinic and University hospital
R5	Female	29	Geriatric fellow	3	University outpatient clinic, University hospital, and veteran hospital
R6	Female	29	Geriatric fellow	3	University outpatient clinic and University hospital
R7	Female	62	Geriatrics	33	Private clinic
E2	Female	55	Geriatrics	25	Private clinic and University hospital
E3	Male	32	Geriatrics	2	Private clinic
W1	Male	52	Family medicine	26	Private clinic

### Quantitative Findings From the uMARS

PCPs positively rated the objective quality of the MGAT web application, with a mean objective quality score of uMARS of 4.06 (SD 1.05; see Table 4). The quality, credibility, and quantity of information were rated the highest (mean 4.60, SD 0.51), while the engagement received the lowest rating (mean 3.34, SD 1.51). The subjective quality rating of MGAT was also moderately high (mean 3.93, SD 1.19), with the item “Recommend MGAT to others” rated highest (mean 4.70, SD 0.48) and the item “Willing to pay for the application” rated the lowest (mean 3.11, SD 1.90).

PCPs scored the highest on perceived impact of MGAT (mean 4.82, SD 0.39). The scores were similar in all aspects, indicating a consistent degree of high perceived impact of MGAT on PCP knowledge, attitudes, and intentions to use MGAT to provide AT services to their patients.

**Table 4:** Scores on the uMARS^a^ scales and subscales for the MGAT web application.

uMARS scales and subscales		Score, mean (SD)
**MGAT^b^ objective quality**		4.06 (1.05)
	Engagement	3.34 (1.51)
	Functionality	4.25 (0.71)
	Aesthetic	4.27 (0.64)
	Information	4.60 (0.51)
**MGAT subjective quality**		3.93 (1.19)
	Recommend the app to others	4.70 (0.48)
	Predicted frequency of use of the app in the next year	4.20 (0.92)
	Willing to pay for the app	3.11 (1.90)
	Overall MGAT rating	3.60 (0.70)
**Perceived impact of MGAT**		4.82 (0.39)
	Awareness	4.60 (0.52)
	Knowledge	4.80 (0.42)
	Attitudes	4.90 (0.32)
	Intention to change	4.90 (0.32)
	Help-seeking	4.70 (0.48)
	Behavior change	5.00 (0.00)

^a^uMARS: User Version of the Mobile Application Scale.

^b^MGAT: Mi Guía de Asistencia Tecnológica (My Assistive Technology Guide).

### Qualitative Insights From Participant Feedback

In this section, a summary of the interview data results is reported, along with representative quotes that provide deeper insight into the reasons behind their scores on each uMARS scale: engagement, functionality, aesthetic, information, subjective quality, and perceived impact.

#### Engagement

PCPs agreed that the MGAT lacks clear customization options, which limited their engagement with the MGAT. This sentiment was expressed by a geriatrician who noted, “I didn’t make any changes to the MGAT because it doesn’t allow it” (E2). In addition, the MGAT was found to lack user interactivity, specifically in terms of input and feedback mechanisms. A geriatric fellow highlighted this issue, stating, “It does not allow me to interact with the application or provide feedback” (R6), which indicates a perceived gap in interactive features. Another geriatric fellow also pointed out the absence of notifications, commenting, “Regarding the alerts, I understood them as notifications, and that is not something the application is currently capable of doing” (R5).

However, all of the participants found the MGAT to be entertaining and interesting, and targeted to older Hispanics. A geriatrician stated, “I loved the part about where you can buy the equipment. I found the videos really cool, they made me smile. And the actors (older Hispanics) were professional” (E2). This PCP also found the MGAT videos appropriate to support older adults learning about AT use: “The application is very valuable because older adults learn with the demonstration.”

#### Functionality

PCPs found the application easy to use, learn, and navigate, as stated by a geriatrician: “The reality is that the application is quite easy to use. I think it is intuitive in that sense (R5).” However, some PCPs noted that scanning the QR code of the MGAT address was challenging for certain patients: “Many of the elderly, unfortunately, get frustrated when using the camera (on the phone) and cannot find the link. And pressing it is difficult for them (E3).” To address this challenge, an internal medicine physician (R2) applied various strategies, such as downloading the MGAT to patients’ cell phone home screens, teaching caregivers how to access and use the MGAT, or using the MGAT from the PCP computer screen.

Although performance received a slightly lower score, PCPs identified issues primarily related to the MGAT’s dependency on a reliable internet connection. This dependency resulted in slow logins or delays in loading MGAT screens and videos. As one internal medicine physician mentioned, “If there is no Internet, it doesn’t work. It has happened to me on the phone that it took me a long time to get help (from the MGAT) for the patient on the phone (R2).” This PCP addressed the internet connection issue by stating: “I downloaded the application on the computer and taught them how to navigate the page without having the problem of a bad signal.”

#### Aesthetics

Regarding aesthetics, the MGAT’s design was praised for its visual appeal. A geriatrician remarked, “I was surprised by the quality of the videos and the information (R5).” Another geriatrician appreciated the MGAT’s layout for diverse users by stating:

For me, it was a good way to divide it, doing it by needs, an easier way to access the options available, not just to a doctor but also for the patient to use it… The app has more options and a wider range of items, so if you were searching for a specific walker, you would be limiting yourself to that. In contrast, if you select that the limitation is mobility, you will see other options you might not have seen.

R6

However, participants provided several suggestions to enhance the MGAT’s aesthetics. Regarding visual appeal, a geriatrician suggested: “Maybe make it more attractive in terms of the application’s visibility… Maybe something more eye-catching in terms of aesthetics… more inviting to use. It looks a little bit analog” (E3). Another internal medicine physician recommended improving the MGAT color scheme: “I think sometimes you need to add more clarity... the contrast can be improved” (R4). In addition, 2 other PCPs recommended increasing the size of some of the MGAT’s elements, with a participant stating, “What could be done is to put the icons larger on the first page... because they need to see the image that is very good” (E3). In this regard, this geriatrician proposed the following strategy to improve visual access to the MGAT: “When you open it from the computer, it can be viewed more completely.”

#### Information Quality

The PCPs agreed that the quality of the information provided by the MGAT was high, as exemplified by this geriatrician’s comment: “I was surprised by the quality of the information” (R5). They also highlighted the relevance of the information to older adults. A family doctor stated, “It has much relevant information regarding what can be offered to patients to help them carry out their independent activities, as well as their safety and fall prevention” (W1). The positive reception of the MGAT by older adults further supports the relevance and appropriateness of the MGAT’s information for this demographic, as illustrated by this internal medicine physician's comment: “It helped patients clarify what equipment they need” (R1).

Regarding the quantity of information, the PCPs appreciated the conciseness of the MGAT, as it avoids overwhelming users with excessive text and facilitates ease of use and teaching. This was expressed by an internal medicine physician who said: “The good thing is that it’s short, meaning it’s not overwhelming to the point I would run out of time to show it or review it” (R1). PCPs also rated the comprehensiveness of the MGAT information highly. However, several PCPs suggested that the MGAT could benefit from more specialized information for medical professionals. Some recommended including specific *ICD-10 (International Statistical Classification of Diseases, Tenth Revision)* diagnostic codes from the Centers for Medicare & Medicaid Services (CMS), as mentioned by this geriatrician (E2):

Many problems we face are that when we place these orders for medical equipment so patients can benefit from their health plans, they ask us for specific codes. In other words, we have to dance to the tune of the health plan. In this regard, the application could also help us. For example, for a cane, what diagnosis code could be effective for getting it approved?

E2

Several PCPs also recommended adding the Healthcare Common Procedure Coding System codes for the devices covered by Medicare to the MGAT. A geriatrician expressed this idea by stating: “You should include, if possible, a more private section for the area of physicians that requires a subscription to access the part of the application that includes the codes (for AT devices)” (E3). Several PCPs also emphasized the importance of including links to CMS information to justify medical devices to insurance plans. A geriatrician stated:

It would be ideal if it took you to the CMS page because you know what? It tells you how to justify the equipment. As a geriatrician, I know how to justify it, but a general practitioner or an internist has no idea how to justify an electric wheelchair for someone who has the use of their hands but whose body does not function.

R3

In addition, 2 PCPs suggested that integrating a questionnaire within the MGAT could streamline the process for PCPs, making it more efficient and likely to increase the MGAT’s usage. This was expressed by an internal medicine physician:

If the application provides a functional problem questionnaire linked to device recommendations, it will help increase the application’s usage compared to a primary doctor simply asking random questions. This way, the doctor won't have to return to the application or search for another educational tool to make recommendations. Having both functions in one place is much better because it reduces the time needed for the task.

R1

Some PCPs also expressed the need for the MGAT to facilitate the transfer of information to patients by sending or printing the information directly from the web application: “Another thing is if it were possible to send a link or print information from the application for the patient to take with them” (R1).

Regarding visual information, participants agreed that the overall clarity of the MGAT photos and videos is satisfactory, as noted by a geriatrician: “I think the photos look clear” (E3). However, to improve the clarity of the visuals for older adults, this participant highlighted the strategy of accessing the MGAT on a computer rather than a mobile device.

A recurring theme was the need for audio instructions to accompany visual content. An internal medicine physician expressed that “The MGAT videos need audio to show them (older adults) how to do it (use the AT device)” (R2), suggesting that auditory cues could enhance older adults’ understanding and retention of information. To further improve the visual explanation in the videos, a PCP also recommended adding subtitles, in addition to audio, stating, “not only narrative but also include the subtitles” (E3).

#### Subjective Quality

PCPs indicated a strong endorsement of the usability of MGAT, evidenced by a high likelihood of recommending it to other physicians. For example, a geriatrician considers the MGAT a valuable tool that can significantly benefit patients, implying a high probability of suggesting it to fellow doctors:

This app has great value because many times we are the first line of contact with the patient who truly needs it… so the more doctors who can have it, the more accessible assistive technologies will become to the community.

E3

Another internal medicine physician suggested that the MGAT benefits other medical specialists as well: “I believe that this is not only directed at primary care doctors but also at physiatrists and rheumatologists” (R4).

Similarly, the willingness of PCPs to use MGAT in the next 12 months, despite time constraints, suggests that the participants view the web application as a useful tool. An internal medicine physician stated, “I put the number 3 to 10 (times that I would use MGAT in the next 12 months) because I depend a lot on time. Sometimes, if I’m busy, I forget to use it” (R2).

The data showed mixed views on paying for the MGAT. Although some PCPs are willing to consider paying for it, others expressed concerns about the cost, particularly from the perspective of older Hispanics who might find it difficult to afford. A geriatrician pointed out: “The ideal situation would be for the application to be free so patients could access it” (E2). This feedback correlates with a lower mean rating of 3.2.

#### Perceived Impact

PCPs reported that the MGAT has significantly heightened their awareness of the functional needs of their patients' daily activities. An internal medicine physician mentioned:

Using the app and being able to show them to the patients has helped me become a little more aware and attentive to ask them more specific questions about their daily lives rather than if they have fallen or are wetting themselves. One asks about things that affect their daily activities, but not necessarily about whether they have problems brushing their teeth, bathing, dressing, putting on a shirt, and with this (the MGAT), I would come and say, ‘Look, to button your shirt, this device helps you,’ and I would show them the buttoner.

R2

All PCPs also expressed that, while they were previously familiar with common AT devices like walkers and canes, the MGAT has expanded their knowledge of other useful devices. A geriatrician commented:

Even though we doctors know these items exist, we are not ‘up to date’ with many of them. So, I learned about many items that I didn’t know existed, and that could help my patients in their daily tasks. Thus, the MGAT helps us to give our patients more options.

R6

PCPs also expressed that the MGAT has significantly increased their attitudes and intentions to facilitate access to AT for their patients. A geriatrician emphasized that the application is highly valuable because it makes them a crucial point of contact for patients needing AT. He stated, “Many times, we are the first line of contact with the patient who really needs it... the more doctors have it, the more accessible AT will be to the community” (E3).

The use of the MGAT also prompted changes in all PCPs’ behaviors regarding informing, demonstrating, and prescribing AT devices to their patients or informing their family members. PCPs found it beneficial to use the MGAT during consultations to visually demonstrate the AT devices, which helped make informed decisions about what might be useful for the patients. As a participant stated:

I showed them how to access it from their cell phone, but to speed up the interaction during the visit, I would say, “When you tap here on the cell phone, this will appear.” I would select any device and usually explain that there would be a description of the different devices they could use. Then, I would show them the video. For some, I would download the app to their cell phones and show them where they could find the devices. I would tell them that if they needed it, I could order it for them, and always mentioned that for the next time (follow-up visit), if they found something they needed, I could order it for them.

E3

A geriatrician talked about the value of the MGAT in increasing her patients’ quality of life.

My job is not only about health but also about quality of life. For them to be able to do gardening with something they wouldn’t consider… such as a gardening chair, and for me to be able to say, “Look, you can do it with this device,” it continues to give them a reason to be alive. … not just to be independent, but to enjoy life. Now with the device, they can dress themselves and go to the opera. … So, having things that give them independence is like an antidepressant.

R3

However, the limited time available during consultations was a significant barrier to using the MGAT. The high volume of patients and the need to prioritize immediate medical concerns often left little time for detailed discussions about assistive technology. A geriatrician mentioned:

In medical appointments, we sometimes have many patients scheduled, and although I would like to show them the video and all the assistive devices' instructions, I didn't have much time with them then. But I had already had the opportunity to see them beforehand, so I knew to whom it applied and to whom it didn't, so I gave them a brief summary of how to use it.

R6

PCPs provided several strategies to manage the time barrier associated with using the MGAT. One strategy was to provide patients access to the MGAT before the medical appointment, allowing them to familiarize themselves with the AT devices and identify their needs during the appointment. Another approach was introducing the MGAT to patients during the medical visit and asking them to identify their specific AT needs for the next follow-up appointment. Finally, several PCPs recommended including a screening of functional abilities in daily activities within the MGAT that could be completed by the patient, their family member, the receptionist, the clinic nurse, or the physician themselves as a strategy for more efficient use of the MGAT. For example, an internal medicine physician explained:

If the application provides you with a questionnaire on functional problems, where one marks the different issues the patient has, which is then linked to a recommendation for a type of assistive device, it will help increase the use of the application instead of a primary care doctor simply asking random questions... If both things are in one place, that's much better because you reduce the working time.

R1

#### Integration of Quantitative and Qualitative Data

Integrating uMARS ratings with qualitative feedback provides a comprehensive understanding of user experiences. For example, the low engagement score correlates with participant comments emphasizing the low engagement score correlates with participant comments that emphasize the lack of customization and interactive elements in the MGAT. Conversely, the higher functionality score aligns with user opinions regarding how easy the MGAT is to use, learn, and navigate.

Furthermore, integrating the uMARS Perceived Impact scores with qualitative feedback provides a comprehensive understanding of the app’s potential to influence knowledge, awareness, and behavior. The high mean score of 4.1 aligns with participants’ comments about increased knowledge and motivation for change.

However, the qualitative data also highlight the need for additional features and support to overcome barriers to sustained behavior change. This insight suggests areas for improvement in the app’s design and functionality to enhance its long-term impact on users’ health behaviors.

## Discussion

### Principal Findings

The primary objective of this study was to assess the quality of the MGAT web application among PCPs and to describe their experiences using it to facilitate access to AT services for older adults. This research is significant as it addresses a critical gap in the provision of AT services for older adults, particularly within Latino populations in Puerto Rico, who often face barriers to accessing necessary devices due to a lack of information and resources [7]. By equipping PCPs with this knowledge, the study aims to improve the provision of AT services, ultimately benefiting the daily functioning, well-being, and quality of life of older adults.

The results indicated that PCPs rated MGAT positively, with a high or moderately high mean score on all dimensions of uMARS, reflecting high perceived quality, usability, and impact on their practice. While the MGAT provided high-quality content, engagement scores were influenced by the low ratings in the uMARS customization item (mean 1.40, SD 0.70) and interactive item (mean 2.20, SD 1.03). The absence of customization options and interactive elements, such as user input, feedback mechanisms, and notifications, was perceived as a limitation. The implications of the lower engagement score for the overall user experience of the MGAT suggest potential areas for improvement in user interaction and customization design. Interactive enhancements, such as allowing users to provide input, receive feedback, and receive notifications about new AT devices, along with customization features like selecting the most commonly prescribed AT devices, can improve engagement while maintaining high information quality.

Despite the overall perceived high quality of the MGAT, several key challenges emerged that impacted its usability, including reliance on a stable internet connection, the absence of audio descriptions for instructional videos on AT use, and the lack of current procedural terminology codes for AT devices, along with their medical justification. These challenges affect PCPs’ ability to effectively use the application, as they may need to employ additional strategies to assist their patients, such as using the MGAT from a computer screen with a wired connection to the internet or spending extra time describing the use of the AT devices to the patients.

The variation in the use of the web application, ranging from 0 to 30 patients in a month among the 10 participating PCPs, highlights differences in engagement and feasibility within clinical workflows. This disparity suggests that while some PCPs were able to integrate the web application consistently into their practice, others faced barriers such as time constraints, competing clinical demands, or varying levels of familiarity with AT tools. Notably, one PCP did not use the MGAT web application at all, which underscores potential challenges in adoption.

These differences in usage may impact the generalizability of the findings, as the benefits and usability perceptions of the web application may be more reflective of those who used it frequently. Furthermore, the wide range in usage suggests that implementation strategies should consider tailored training, workflow integration support, and additional usability enhancements to encourage more consistent adoption among PCPs.

### Comparison With Previous Work

The findings of this study are consistent with existing literature, which shows that health care providers are more likely to adopt technology that is intuitive, easy to navigate, perceived as useful, and encourages them to use it [36]. Similar to previous studies, this research highlights the need for user-friendly designs to ensure the effective use of health technology [37]. However, our study contributes new insights by highlighting specific areas for improvement, such as improving engagement and addressing technical issues, which are less frequently discussed in the literature.

Although there is a web-based AT tool, other than the MGAT, to improve access to AT information among people with disabilities—the Atvisor [38]—both tools represent 2 distinct approaches, each catering to the needs of different users. MGAT is specifically designed for older Latinos in Puerto Rico, providing user-friendly access to information on AT devices in various daily activities. Its simplicity and visual aids make it particularly accessible to older adults, enhancing awareness among PCPs about functional needs and available solutions. In contrast, Atvisor.ai is a clinical decision support system aimed at AT professionals and users in Israel, allowing personalized assessments based on individual needs and goals. This platform incorporates advanced features such as a matching algorithm to connect users with suitable products, facilitating online purchases and long-term follow-up. However, the strengths of MGAT lie in its ease of use and culturally relevant content, which is particularly beneficial for older adults who may be less familiar with technology.

Our observations regarding the differences between MGAT and Atvisor are based on an experiential, hands-on comparison, rather than an empirical research study. While MGAT’s design prioritizes simplicity for PCPs requiring immediate access to AT information without complex decision-support features, Atvisor offers a more intricate system for environments where detailed evaluations and ongoing support are necessary. To avoid overclaiming, we acknowledge the need for future comparative studies to validate these distinctions empirically, particularly in terms of usability, user workload, and task efficiency.

This study holds significant value for PCPs who work with older adults. By providing a comprehensive resource for AT options, the MGAT can enhance patient care by allowing physicians to make informed recommendations tailored to their patients' functional needs, thus boosting the independence and quality of life of older adults. PCP recommendations for app improvements, such as integrating diagnostic codes and adding audio instructions, highlight areas where further development could increase the adoption and utility rates among health care providers.

### Limitations and Future Research

This study acknowledges several limitations that may affect the interpretation of the results. The sample size was relatively small and consisted primarily of geriatricians, which may not fully represent the broader spectrum of PCPs. Furthermore, the study was conducted in a specific geographical and cultural context, which may limit the generalizability of the findings. The use of purposive sampling and snowball methods may introduce bias. These methods, while effective for identifying participants with specific experiences, can lead to a sample that is not representative of the broader population. We acknowledge this potential bias as a limitation of our study and suggest that future research consider more diverse sampling strategies to enhance generalizability. Future efforts should explore strategies to streamline app use within time-constrained environments, such as optimizing interface efficiency or integrating the tool into existing electronic health record systems. Future studies should also use a more diverse sample of health care providers with varied workload constraints to explore the usability of the app in different settings. Future iterations of the MGAT should focus on addressing the technical challenges identified in this study and incorporate user-centered design approaches to optimize both usability and engagement, ensuring a more holistic user experience. Further studies could also explore the long-term impact of MGAT on functional outcomes of patients and investigate the effectiveness of the app in other cultural contexts. Finally, research could examine the integration of MGAT with other disability services in primary care to provide a more comprehensive solution for the management of FDs among older adults.

### Conclusions

PCPs positively rated the MGAT web application, with a high perceived impact on their practice. MGAT shows promise as a valuable tool for PCPs in Puerto Rico, offering comprehensive and culturally relevant information on AT devices. Although technical challenges must be addressed, the positive reception of MGAT highlights its potential to improve the provision of assistive technology services for older adults. By improving PCP knowledge and awareness of AT options, MGAT can contribute to better patient care and improved outcomes for function, health, and quality of life for older adults.

## Abbreviations

ADL: activities of daily living
AT: assistive technology
CMS: Centers for Medicare & Medicaid Services
FD: functional disability
ICD-10: International Statistical Classification of Diseases, Tenth Revision
MGAT: Mi Guía de Asistencia Tecnológica (My Assistive Technology Guide)
PCP: primary care physician
PI: principal investigator
uMARS: User Version of the Mobile Application Rating Scale

## Data Availability

The datasets generated or analyzed during this study are available from the corresponding author upon reasonable request.

## References

[1] Ben Mortenson W, Demers L, Fuhrer MJ, Jutai JW, Bilkey J, Plante M, DeRuyter F (2018). Effects of a caregiver-inclusive assistive technology intervention: a randomized controlled trial. BMC Geriatr.

[2] Stanley R (2015). Technology supports for community-dwelling frail older adults. ar.

[3] Yusif S, Soar J, Hafeez-Baig A (2016). Older people, assistive technologies, and the barriers to adoption: A systematic review. Int J Med Inform.

[4] Chong N, Akobirshoev I, Caldwell J, Kaye HS, Mitra M (2022). The relationship between unmet need for home and community-based services and health and community living outcomes. Disabil Health J.

[5] Wilson DJ, Mitchell JM, Kemp BJ, Adkins RH, Mann W (2009). Effects of assistive technology on functional decline in people aging with a disability. Assist Technol.

[6] Szanton SL, Leff B, Wolff JL, Roberts L, Gitlin LN (2016). Home-based care program reduces disability and promotes aging in place. Health Aff (Millwood).

[7] Orellano-Colón EM, Rivero-Méndez M, Ralat-Fonseca BN, Varas-Díaz N, Lizama-Troncoso M, Jiménez-Velázquez IZ, Jutai JW (2024). Multilevel barriers to using assistive technology devices among older hispanics from poor and disadvantaged communities: the relevance of a gender analysis. Disabil Rehabil Assist Technol.

[8] Prajapati G, Sharmila K (2023). Difficulties experienced by older adults when not using assistive devices. Discov Soc Sci Health.

[9] Al‐Oraibi S (2012). Impact and economic assessment of assistive technology in care homes in Norfolk, UK. J Assist Technol.

[10] Meiland F, Innes A, Mountain G, Robinson L, van der Roest H, García-Casal J A, Gove D, Thyrian JR, Evans S, Dröes RM, Kelly F, Kurz A, Casey D, Szcześniak D, Dening T, Craven MP, Span M, Felzmann H, Tsolaki M, Franco-Martin M (2017). Technologies to support community-dwelling persons with dementia: A position paper on issues regarding development, usability, effectiveness and cost-effectiveness, deployment, and ethics. JMIR Rehabil Assist Technol.

[11] Waldron D, Layton N (2008). Hard and soft assistive technologies: defining roles for clinicians. Aust Occup Ther J.

[12] (2020). Healthy ageing and functional ability internet. World Health Organization.

[13] Orellano-Colón EM, Ramos-Marichal AI, González-Crespo VR, Zeballos-Hernández BN, Ruiz-Márquez KN, Roche-Lima A, Adorno-Mercado JM, Laborde-Torres NA, Berríos-Llopart JG, Cruz-Ramos AM, Montenegro DV, Lamoutte CE, Rosa-Casilla ND, Meléndez-Berrios DE (2024). Breaking barriers: the design and development of an assistive technology web app for older latinos with disabilities in daily activities. Technologies (Basel).

[14] Orellano-Colón EM, Rivero-Méndez M, Lizama M, Jutai JW (2018). Assistive technology unmet needs of independent living older Hispanics with functional limitations. Disabil Rehabil Assist Technol.

[15] Orellano-Colón EM, Suárez-Pérez EL, Rivero-Méndez M, Boneu-Meléndez CX, Varas-Díaz N, Lizama-Troncoso M, Jiménez-Velázquez IZ, León-Astor A, Jutai JW (2021). Sex disparities in the prevalence of physical function disabilities: a population-based study in a low-income community. BMC Geriatr.

[16] Orellano-Colón EM, Fernández-Torres A, Figueroa-Alvira N, Ortiz-Vélez B, Rivera-Rivera NL, Torres-Ferrer GA, Martín-Payo R (2024). Empowering potential of the my assistive technology guide: exploring experiences and user perspectives. Disabilities (Basel).

[17] Martin Payo R, Fernandez Álvarez M M, Blanco Díaz M, Cuesta Izquierdo M, Stoyanov S, Llaneza Suárez E (2019). Spanish adaptation and validation of the mobile application rating scale questionnaire. Int J Med Inform.

[18] Johnson SG, Potrebny T, Larun L, Ciliska D, Olsen NR (2022). Usability methods and attributes reported in usability studies of mobile apps for health care education: scoping review. JMIR Med Educ.

[19] Deniz-Garcia A, Fabelo H, Rodriguez-Almeida AJ, Zamora-Zamorano G, Castro-Fernandez M, Alberiche Ruano MDP, Solvoll T, Granja C, Schopf TR, Callico GM, Soguero-Ruiz C, Wägner AM, WARIFA Consortium (2023). Quality, usability, and effectiveness of mHealth apps and the role of artificial intelligence: current scenario and challenges. J Med Internet Res.

[20] Manzano-Monfort G, Paluzie G, Díaz-Gegúndez M, Chabrera C (2023). Usability of a mobile application for health professionals in home care services: a user-centered approach. Sci Rep.

[21] Fetters MD, Curry LA, Creswell JW (2013). Achieving integration in mixed methods designs-principles and practices. Health Serv Res.

[22] Karagianni K Optimizing the UX honeycomb—A small amendment to the classic diagram hopefully improves its UX.

[23] Morville P User experience design. Semantic Studios.

[24] Scherer MJ (2005). Living in the State of Stuck: How Technology Impacts the Lives of People with Disabilities.

[25] Kasym M (2022). How to make the older users love your product examples of UX design for seniors. Eleken.

[26] Nielsen J (2013). Usability for senior citizens: improved but still lacking. NielsenNormanGroup.

[27] Kane L (2019). Usability for senior citizens: challengers and changes. NielsenNormanGroup.

[28] Bryant JA, David P (2020). Solving for inclusive technology for older adults. AARP International.

[29] Trzepla S (2019). UX accessibility for elderly: 12 principles. UX Planet.

[30] Hwang W, Salvendy G (2010). Number of people required for usability evaluation. Communications of the ACM.

[31] Nielsen J, Landauer T (1993). A mathematical model of the finding of usability problems.

[32] Kushniruk AW, Patel VL (2004). Cognitive and usability engineering methods for the evaluation of clinical information systems. J Biomed Inform.

[33] Kadam PD, Chuan HH (2016). Erratum to: rectocutaneous fistula with transmigration of the suture: a rare delayed complication of vault fixation with the sacrospinous ligament. Int Urogynecol J.

[34] Stoyanov SR, Hides L, Kavanagh DJ, Wilson H (2016). Development and validation of the user version of the mobile application rating scale (uMARS). JMIR Mhealth Uhealth.

[35] Kibiswa N (2019). Directed qualitative content analysis (DQlCA): A tool for conflict analysis. TQR.

[36] Borges do Nascimento IJ, Abdulazeem H, Vasanthan LT, Martinez EZ, Zucoloto ML, Østengaard L, Azzopardi-Muscat N, Zapata T, Novillo-Ortiz D (2023). Barriers and facilitators to utilizing digital health technologies by healthcare professionals. NPJ Digit Med.

[37] Kumar S, Nilsen WJ, Abernethy A, Atienza A, Patrick K, Pavel M, Riley WT, Shar A, Spring B, Spruijt-Metz D, Hedeker D, Honavar V, Kravitz R, Lefebvre RC, Mohr DC, Murphy SA, Quinn C, Shusterman V, Swendeman D (2013). Mobile health technology evaluation: the mHealth evidence workshop. Am J Prev Med.

[38] Ran M, Banes D, Scherer MJ (2022). Basic principles for the development of an AI-based tool for assistive technology decision making. Disabil Rehabil Assist Technol.

